# Vascularization, Innervation, and Inflammation: Pathways Connecting the Heart–Brain Axis and Implications in a Clinical Setting

**DOI:** 10.3390/biomedicines13010171

**Published:** 2025-01-13

**Authors:** Alexa R. Lauinger, Joseph J. Sepe

**Affiliations:** 1 Department of Biomedical and Translational Sciences, Carle Illinois College of Medicine, University of Illinois at Urbana-Champaign, Urbana, IL 61801, USA; alexa5@illinois.edu; 2 Department of Integrative Biology and Physiology, University of Minnesota Medical School, Minneapolis, MN 55455, USA

**Keywords:** heart–brain axis, cardiovascular disease, stroke, autonomic neuromodulation

## Abstract

With an aging population, the incidence of both ischemic heart disease and strokes have become the most prevalent diseases globally. These diseases have similar risk factors, such as hypertension, diabetes, and smoking. However, there is also evidence of a relationship between the heart and the brain, referred to as the heart–brain axis. In this relationship, dysfunction of either organs can lead to injury to the other. There are several proposed physiologies to explain this relationship. These theories usually involve vascular, neuromodulatory, and inflammatory processes; however, few articles have explored and compared these different mechanisms of interaction between the heart and brain. A better understanding of the heart–brain axis can inform physicians of current and future treatment and preventive care options in heart and brain pathologies. The relationship between the brain and heart depends on inflammation, vascular anatomy and function, and neuromodulation. The pathways connecting these organs often become injured or dysfunctional when a major pathology, such as a myocardial infarction or stroke, occurs. This leads to long-term impacts on the patient’s overall health and risk for future disease. This study summarizes the current research involved in the heart–brain axis, relates these interactions to different diseases, and proposes future research in the field of neurocardiology. Conditions of the brain and heart are some of the most prevalent diseases. Through understanding the connection between these two organs, we can help inform patients and physicians of novel therapeutics for these pathologies.

## 1. Introduction

As the age of the population increases, there has also been an increase in the incidence of ischemic heart disease and strokes. These events account for the top two causes of death globally [1,2]. The risk factors for cardiovascular disease and stroke are similar, including hypertension, smoking, and diabetes [3,4]. Evidence suggests that following a myocardial infarction, patients have an increased risk of a stroke compared to the general population [5]. These relationships are indicative of a heart–brain axis where injury to either organ can lead to dysfunction or impairment of the other, and the study of this interplay has grown into the field of neurocardiology [6].

Within neurocardiology there are multiple theories exploring how the brain and heart can impact each other and the connection between them in different disease states. One of the proposed mechanisms of influence involves the changes in the gut microbiome related to the brain and to the heart that impact one another [7]. More approaches look at the direct interactions between these organs through vascularization, innervation, and inflammation [8,9,10]. Evidence of these different theories has been tested through various cell-to-cell interactions and the use of animal models [11,12,13]. These mechanisms have been explored in a range of different acute and chronic diseases, including stroke, atrial fibrillation, and depression [14,15,16]. As an example, the neurological manifestations of structural and rheumatic heart disease include syncope, migraine, and seizures [17,18].

In understanding the relationship between the heart and brain in these diseases, we can begin to extrapolate this knowledge into other neurological or cardiac conditions and the interplay between them. It can also help shape treatments and therapies that extend beyond a single organ or disease. In this field, there has been several discussions of the innervation and inflammatory pathways that affect the heart and brain; however, there is a lack of discussion around the clinical application and treatment opportunities for these relationships. Further exploration into the prior results regarding the physiology and treatment outcomes will offer an opportunity to develop interventions. Therefore, the purpose of this review is to summarize the current understanding of the heart–brain axis through vascularization, innervation, and inflammation, to explore the application of these mechanisms to the development of emerging therapies and propose ways that this perspective can inform best treatment for neurological and cardiovascular conditions. The authors also propose relationships in the heart–brain axis that are poorly studied, such as mood disorders and trigeminal neuralgia.

## 2. Biology of the Heart–Brain Axis

**Figure 1:**
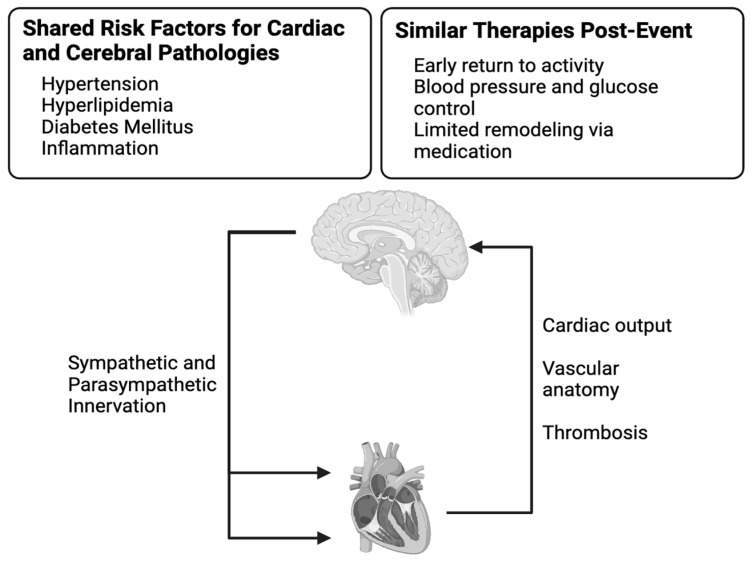
Overview of the pathways in the heart–brain axis.

Neurons and cardiac myocytes are unique types of cells with excitable membranes that both utilize electrical signals to alter their function or communicate with nearby cells, and as a result these systems are in constant communication to regulate each other [19]. This allows the need for blood to the brain to be balanced by changes in cardiac output and vascular resistance. In addition to the neuromodulation and vasculature of the heart–brain axis, inflammation connects these two organs and their pathologies (Figure 1).

### 2.1. Innervation in the Heart–Brain Axis

Autonomic regulation of the cardiac system plays a key role in the development and recovery of cerebrovascular disease. The sympathetic and parasympathetic branches of this system regulate heart rate, contractility, and vascular tone [20,21]. A balance between these two systems is required, and imbalance can result in lethal dysrhythmias [22]. Damage to the cardiac nervous system can occur through neuropathy, especially in the setting of diabetes mellitus, ischemic injury, or during a medical intervention such as cardiac transplant [20]. In response, cardiac resynchronization therapy is used in heart failure to improve overall function [23]. However, the innervation system of the heart has plasticity due to structural and neurochemical changes that occur after injury. Increased growth and proliferation of sympathetic nerves can lead to the development of arrythmias, especially after myocardial infarctions, hypertension, or heart failure [21].

Apart from neuronal growth, neurohumoral transmission is a major regulator of heart function from the neurons that control the cardiac system. Catecholamines are responsible for regulating the cardiac contractility and heart rate while overstimulation of this pathway can lead to heart failure [24]. Diseased neurons in the cardiac innervation system lead to disruption of the neurotransmitter network. In this setting, excessive noradrenergic and neuropeptide Y release cause microvascular constriction and lead to myocardial infarctions, heart failure, and arrythmias [25]. Based on the innervation and neurohumoral control of the cardiac system, growth factors and regulatory mechanisms have been proposed as potential therapeutics following cardiac injury [22].

### 2.2. Vascular Influence of the Brain

The brain does not have its own energy storage and therefore requires a constant, regulated blood supply [26]. It receives its blood supply from the neurovasculature, and this allows the transport of glucose to areas of the brain that require higher expenditures of energy [8]. The arterial blood supply of the brain arises from the internal carotid and vertebral arteries [27]. The role of neurovasculature has become an important factor in understanding a number of cerebellar diseases, with the most notable being stroke, but there is also evidence of its impact in migraines, epilepsy, and neurodegenerative diseases [8,28].

Cerebral blood flow is controlled through autoregulation by neurotransmitter release, tone of the neurovasculature, metabolic mechanisms, and endothelial response [29,30]. These mechanisms allow centers of the brain to detect changes in the pH of the blood, as a marker of carbon dioxide levels and changes in intracranial pressure related to blood pressure [31]. After detecting these changes, astrocytes, neurons, and endothelial cells have the ability to release vasoactive transmitters to alter the cerebral blood flow [30]. The brain also, through efferent autonomic pathways, modulates the heart rate, contractility of the ventricles, and peripheral vascular tone in order to regulate blood pressure and direct systemic blood flow appropriately [32]. If any of these pathways fail then there is a risk of decreased blood flow to the brain and resulting pathology.

Resting cerebral blood flow varies with age and sex. Peak blood flow velocity declines with age and is greater in females [33]. Alterations in cerebral blood flow have been suggested in the pathophysiology of neurologic disease, such as Alzheimer’s and ischemic stroke [34,35]. Decreased flow can lead to diminished cellular energy and death [36]. This pathway may indicate that therapies directed at increasing cerebral blood flow could improve cognitive function [37]. There have also been strong associations between variations in cerebral blood flow and migraines, traumatic brain injuries (TBIs), and depression [38,39,40]. Variations in the location of high and low blood flow rate in the brain have been associated with the onset and severity of migraines [38]. Pharmaceutical regulation of vasodilation has been used as a treatment for migraines [41]. In TBIs, hypotension and hypoxia lead to further deterioration and decreased cerebral blood flow. This is supported by evidence that evaluation of the vascular system correlates with overall outcomes [39]. Inability to regulate cerebral blood flow has been associated with depression, including changes in oxidative stress, activation of the hypothalamic–pituitary–adrenal axis, and cytokine changes [40]. Regulatory ability improved with extended treatment with antidepressants [40].

In addition to the quality of function of the neurovascular, the structure of it also has neurological consequences. Trigeminal neuralgia and intracerebral hemorrhage are both cases where the structure of the neurovascular leads to the pathology. The pathophysiology behind trigeminal neuralgia involves the compression of the trigeminal nerve by vasculature [42]. Treatment for this includes surgical decompression of the nerve [43]. Whereas, intracerebral hemorrhages often occur due to abnormalities in the neurovascular system, especially in cases of arteriovenous malformations (AVMs) [44]. Surgical treatment is required to reduce this risk [45].

### 2.3. Impacts of Systemic Inflammation

Inflammation in an acute setting is an essential biological function in destroying threats to the host and repairing normal function of body tissues; however, chronic inflammation is a known factor of disease initiation and progression [46]. Immune activation is modulated by a balance of pro- and anti-inflammatory cytokines that impact endothelial integrity, tissue repair, and cell homeostasis [46,47,48]. However, there are diseases that arise from chronic inflammation. The effects of a dysregulated immune response have been associated with chronic wounds [49], metabolic syndromes [50], liver and kidney disease [51,52], neurodegeneration [53], heart disease [48], and cancer development and progression [54], along with other chronic diseases. Due to its extensive impacts, recent research has focused on the mechanisms and pathophysiology of systemic and chronic inflammation.

In healthy inflammation, a local injury causes the release of chemical modulators that recruit immune cells to the site and cause vasodilation of nearby vessels [55]. Once the activating stimulus is eliminated, a sequence of modulator cytokines is produced to reduce the inflammation and dampen cell recruitment [56]. These changes encourage the removal of pathogens and cell debris followed by the recovery of the cells in the area. Immune system dysfunction due to uncontrolled pro-inflammatory signals or impaired resolution leads to a sustained inflammation. The activation of pro-inflammatory pathways, such as those influenced by NF-kB, increase oxidative stress and endothelial dysfunction responsible for atherosclerosis progression [47]. Prolonged inflammation also leads to cardiac remodeling and fibrosis through cytokine and matrix metalloproteinase activation [48]. The shift from acute to chronic inflammation has systemic effects in the body. It impacts the homeostasis of immune and metabolic processes, which can lead to a vulnerability to infection and the development of metabolic syndromes [57,58]. Through these mechanisms, the diseases of the heart and brain can impact one another. Several types of heart disease cause a systemic inflammatory response that has been suggested as a method to predict prognosis [59].

The impact of inflammation on the heart and brain is a factor in a wide variety of pathologies. Pro-inflammatory cytokines affect the heart through oxidative stress and calcium mishandling, leading to cellular dysfunction and ultimately cardiac fibrosis [60]. Activation of dendritic cells and T-cells play a role in hypertension through oxidative stress as well [61]. In the brain, microglia are a major regulator of inflammation by the production and release of proinflammatory cytokines. This pathway leads to depression of the neuronal synapses and the weakening of signaling [62]. Neuroinflammation by microglia has also been linked to the aggregation of proteins in the brain associated with Alzheimer’s disease [63].

These physiological changes can lead to ischemic stroke or myocardial infarction from thrombosis, heart failure due to myocardial fibrosis, and neurodegeneration due to cell injury [64,65,66,67]. This is a widespread problem, in part, due to the number of diseases that can lead to chronic inflammation. Apart from infection, cancer, physical injury, chemical exposure, neurological factors, and ischemia can all result in systemic, long-term inflammation [68]. As a result, injury to either the brain or the heart can impact each other through inflammation. There is evidence of an increased risk for heart disease, ranging from QTc prolongation to myocardial infarction, to occur after an incident of stroke [69]. Likewise, strokes are more common in patients that have previously had a heart attack [70].

In this multifactorial problem, lifestyle and environment may play a role in outcomes, but there is evidence of increased risk with injury. As the understanding of the impacts of injury to the heart or the brain improves, there should be a focus on using rehabilitation regiments and medications focused on avoiding injury to other organs in the body.

## 3. Discussion and Therapeutic Opportunities

Autonomic neuromodulation therapies (ANMTs) are an emerging field of therapeutics aimed at modulating the cardiac autonomic nervous system and related pathologies. Other ANMTs include vagus nerve stimulation (VNS), ganglionated plexus ablation, stellate ganglion block, and epicardial injections, which all target neuronal connections along the neurocardiac axis.

VNS has received the highest attention for its research and human therapeutic potential. Several studies over the past two decades have shown that neuromodulation of the autonomic nervous system via electrical VNS has promising clinical applications [71]. Approved by the FDA for treatment of pharmacoresistant depression and epilepsy [72], VNS has become an emerging therapeutic and research topic for the treatment of several conditions including cardiovascular disease [73], chronic inflammatory diseases [74,75], postural orthostatic tachycardia syndrome (POTS) [76], and stroke [77]. VNS can be accomplished by direct stimulation of the vagus nerve, or transcutaneously (tVNS).

Direct stimulation of the vagus nerve, which requires surgical implantation of the device and electrodes, has been found to mitigate post-operative atrial fibrillation and inflammation following cardiovascular surgery [78,79]. As reviewed by Yap et al., although the most effective stimulation site remains unclear, tVNS in the ear regions (auricular branch) or the neck region (cervical branch) has shown promising results in clinical practice [80]. Compared to traditional VNS, this approach offers a non-invasive way to impact conditions of both the brain and the heart through tVNS, does not require a surgical procedure, is widely accessible, and is more cost effective compared to implantable VNS.

Although the exact mechanisms through which VNS confers a therapeutic benefit remain unclear, there is strong evidence that it can modify the inflammatory response [79] and suppress a high sympathetic tone by damaging the left stellate ganglion [81]. This is known as the parasympathetic anti-inflammatory pathway, which is driven by the release of cytokines [82]. This has been suggested as a possible therapy for cardiovascular disease, Alzheimer’s disease, depression, and other neurological conditions [82].

Apart from VNS, new therapeutics are being developed that promote cardiac re-innervation following myocardial infarction [25,83], a process which has been shown to beneficially modify the immune response and decrease arrhythmia susceptibility [84]. Additionally, cardiac innervation can be modulated through the regulation of neurotransmitters. For example, the use of paroxetine as a GRK2 inhibitor has been investigated to regulate remodeling after myocardial infarction [24]. Furthermore, anti-inflammatory treatments, such as Baicalin, improve cardiac remodeling [85]. Anti-inflammatory medications have also been suggested in heart failure to minimize cardiac fibrosis and cell death [86]. In a similar perspective, anti-inflammatory medications have been suggested as a possible prophylactic for vascular events following prior ischemic strokes [87]. Multi-drug targeting regiments have been explored as potential treatments for these neurological diseases, with some evidence of them slowing disease progression in chronic conditions [88]. In Alzheimer’s disease, anti-inflammatory medication has been proposed to increase neuronal survival and phagocytosis of protein amyloid-beta plaques [89]. Anti-inflammatory medication has also been suggested as a potential therapy for depression, with promising results [90].

Neurological conditions stemming from the neurovasculature, such as strokes and migraines, utilize many of the same treatments as cardiac conditions, such as antihypertensives and calcium channel blockers to regulate and protect blood vessels from further injury [91,92]. The vascular pathway of the heart–brain axis also has a few surgical treatments to clear or move the blood vessels leading to the disease. Mechanical thrombosis involves surgical removal of a clot to treat an ischemic stroke [93]. Microvascular decompression is an example of a surgical intervention for the neurovascular pathway that involved the movement of blood vessels away from nerves to reduce the interactions between these structures [94].

The current discussion of treatments regarding the heart–brain axis has a heavy focus on the autonomic regulation of chronic conditions of the cardiology system; however, there is a severe deficit of the treatments that could be used after a neurological or cardiac event, such as a traumatic brain injury or myocardial infarction. In addition, there is a severe lack of investigation into immune regulation for neurological and cardiac disease. Although there has been sufficient evidence linking immune responses to the heart–brain axis, this field is overlooked when discussing potential therapies, especially after acute events. It is necessary for future work to explore the impacts of immunological modulation in the context of the heart–brain axis. One of the largest barriers to new therapeutics in this field is a mixture of inconsistent outcomes, likely due to patient-specific comorbidities and complex cell-to-cell interactions following acute injuries [86,95].

## 4. Conclusions and Future Directions

The study of the heart–brain axis and their impacts in disease is a developing field, and to the point, much of the literature focuses its perspective on stroke and myocardial infarction. Although there is evidence of post event interaction between the two organs, there are also several risk factors that are associated with both ischemic heart disease and stroke. These factors complicate the investigation of the relationship between these two organs. Many of the neuromodulation studies have utilized animal models due to the difficulty to validate dysregulation in humans. As diagnostic tools for neuromodulatory dysfunction evolve, we will have a better understanding and evidence of the relationship between the brain and the heart.

There has been a recent exploration of the systemic impacts of heart disease, and one of the most extensive relationships is between the brain and the heart. This axis is regulated through cardiac innervation, the neurovascular system, and inflammation. These pathways demonstrate a relationship between the pathologies of each organ. Examining the pathways can help inform physicians of long-term health effects and lead to potential therapies for these diseases. The study on neurocardiology and the heart–brain axis is still an undeveloped field, and most of the current research focuses on the interactions in vascular or systemic inflammatory diseases. However, a limited number of studies have shown a potential relationship between the heart and the brain in mood disorders, trigeminal neuralgia, and arrhythmias. Understanding the relationship of these diseases may expand our knowledge on their pathophysiology and improve treatment options and preventative measures. Further research is required in these less common pathologies.

## Data Availability

No new data were created or analyzed in this study. Data sharing is not applicable to this article.

## References

[1] Khan M.A., Hashim M.J., Mustafa H., Baniyas M.Y., Al Suwaidi S.K.B.M., Alkatheeri R., Alblooshi F.M.K., Almatrooshi M.E.A.H., Alzaabi M.E.H., Al Darmaki R.S. (2020). Global Epidemiology of Ischemic Heart Disease: Results from the Global Burden of Disease Study. Cureus.

[2] GBD 2019 Stroke Collaborators (2021). Global, Regional, and National Burden of Stroke and Its Risk Factors, 1990–2019: A Systematic Analysis for the Global Burden of Disease Study 2019. Lancet Neurol..

[3] Zhan C., Shi M., Wu R., He H., Liu X., Shen B. (2019). MIRKB: A Myocardial Infarction Risk Knowledge Base. Database.

[4] Boehme A.K., Esenwa C., Elkind M.S.V. (2017). Stroke Risk Factors, Genetics, and Prevention. Circ. Res..

[5] Johansson S., Rosengren A., Young K., Jennings E. (2017). Mortality and Morbidity Trends after the First Year in Survivors of Acute Myocardial Infarction: A Systematic Review. BMC Cardiovasc. Disord..

[6] van der Wall E.E., van Gilst W.H. (2013). Neurocardiology: Close Interaction between Heart and Brain. Neth. Heart J..

[7] Salvadori M., Rosso G. (2024). Update on the Gut Microbiome in Health and Diseases. World J. Methodol..

[8] Iadecola C. (2017). The Neurovascular Unit Coming of Age: A Journey through Neurovascular Coupling in Health and Disease. Neuron.

[9] Wang M., Pan W., Xu Y., Zhang J., Wan J., Jiang H. (2022). Microglia-Mediated Neuroinflammation: A Potential Target for the Treatment of Cardiovascular Diseases. J. Inflamm. Res..

[10] Yang S., Webb A.J.S. (2023). Associations between Neurovascular Coupling and Cerebral Small Vessel Disease: A Systematic Review and Meta-Analysis. Eur. Stroke J..

[11] Ardell J.L., Andresen M.C., Armour J.A., Billman G.E., Chen P.-S., Foreman R.D., Herring N., O’Leary D.S., Sabbah H.N., Schultz H.D. (2016). Translational Neurocardiology: Preclinical Models and Cardioneural Integrative Aspects. J. Physiol..

[12] Kang Y.-M., Zhang Z.-H., Xue B., Weiss R.M., Felder R.B. (2008). Inhibition of Brain Proinflammatory Cytokine Synthesis Reduces Hypothalamic Excitation in Rats with Ischemia-Induced Heart Failure. Am. J. Physiol. Heart Circ. Physiol..

[13] Crestani C.C., Alves F.H.F., Resstel L.B.M., Corrêa F.M.A. (2007). Cardiovascular Effects of Noradrenaline Microinjection in the Bed Nucleus of the Stria Terminalis of the Rat Brain. J. Neurosci. Res..

[14] Critchley H.D., Harrison N.A. (2013). Visceral Influences on Brain and Behavior. Neuron.

[15] Nistor I.R., Gherasim L. (2023). From Neurocardiology to Stroke-Heart Syndrome. Rom. J. Intern. Med..

[16] Sposato L.A., Gurol M.E. (2021). Advances in Neurocardiology: Focus on Atrial Fibrillation. Stroke.

[17] Vasconcelos L.P.B., da Silva Bastos Vasconcelos M.C., Di Flora F.B.M.E., de Oliveira F.A.P., Lima P.D., Silva L.C.B.E., Mucelli Spolaor B.C., da Silva J.L.P., de Magalhães Esteves W.A., Nunes M.C.P. (2022). Neurological and Psychiatric Disorders in Patients with Rheumatic Heart Disease: Unveiling What Is Beyond Cardiac Manifestations. Glob. Heart.

[18] Guimarães R.B., Essebag V., Furlanetto M., Yanez J.P.G., Farina M.G., Garcia D., Almeida E.D., Stephan L., Lima G.G., Leiria T.L.L. (2018). Structural Heart Disease as the Cause of Syncope. Braz. J. Med. Biol. Res..

[19] Ardell J.L., Armour J.A. (2016). Neurocardiology: Structure-Based Function. Comprehensive Physiology.

[20] Filipović N., Marinović Guić M., Košta V., Vukojević K. (2023). Cardiac Innervations in Diabetes Mellitus—Anatomical Evidence of Neuropathy. Anat. Rec..

[21] Hasan W. (2013). Autonomic Cardiac Innervation. Organogenesis.

[22] Ieda M., Fukuda K. (2009). New Aspects for the Treatment of Cardiac Diseases Based on the Diversity of Functional Controls on Cardiac Muscles: The Regulatory Mechanisms of Cardiac Innervation and Their Critical Roles in Cardiac Performance. J. Pharmacol. Sci..

[23] Jaffe L.M., Morin D.P. (2014). Cardiac Resynchronization Therapy: History, Present Status, and Future Directions. Ochsner J..

[24] Parichatikanond W., Duangrat R., Kurose H., Mangmool S. (2024). Regulation of β-Adrenergic Receptors in the Heart: A Review on Emerging Therapeutic Strategies for Heart Failure. Cells.

[25] Clyburn C., Sepe J.J., Habecker B.A. (2022). What Gets on the Nerves of Cardiac Patients? Pathophysiological Changes in Cardiac Innervation. J. Physiol..

[26] Donnelly J., Budohoski K.P., Smielewski P., Czosnyka M. (2016). Regulation of the Cerebral Circulation: Bedside Assessment and Clinical Implications. Crit. Care.

[27] Sethi D., Gofur E.M., Munakomi S. (2024). Anatomy, Head and Neck: Carotid Arteries. StatPearls.

[28] Reiss Y., Bauer S., David B., Devraj K., Fidan E., Hattingen E., Liebner S., Melzer N., Meuth S.G., Rosenow F. (2023). The Neurovasculature as a Target in Temporal Lobe Epilepsy. Brain Pathol..

[29] Silverman A., Petersen N.H. (2024). Physiology, Cerebral Autoregulation. StatPearls.

[30] Claassen J.A.H.R., Thijssen D.H.J., Panerai R.B., Faraci F.M. (2021). Regulation of Cerebral Blood Flow in Humans: Physiology and Clinical Implications of Autoregulation. Physiol. Rev..

[31] Budohoski K.P., Czosnyka M., Kirkpatrick P.J., Smielewski P., Steiner L.A., Pickard J.D. (2013). Clinical Relevance of Cerebral Autoregulation Following Subarachnoid Haemorrhage. Nat. Rev. Neurol..

[32] Kaufmann H., Norcliffe-Kaufmann L., Palma J.-A. (2020). Baroreflex Dysfunction. N. Engl. J. Med..

[33] Koep J.L., Bond B., Barker A.R., Ruediger S.L., Pizzey F.K., Coombes J.S., Bailey T.G. (2022). The Relationships between Age, Sex, and Cerebrovascular Reactivity to Hypercapnia Using Traditional and Kinetic-Based Analyses in Healthy Adults. Am. J. Physiol. Heart Circ. Physiol..

[34] Kisler K., Nelson A.R., Montagne A., Zlokovic B.V. (2017). Cerebral Blood Flow Regulation and Neurovascular Dysfunction in Alzheimer’s Disease. Nat. Rev. Neurosci..

[35] Biose I.J., Oremosu J., Bhatnagar S., Bix G.J. (2023). Promising Cerebral Blood Flow Enhancers in Acute Ischemic Stroke. Transl. Stroke Res..

[36] Roher A.E., Debbins J.P., Malek-Ahmadi M., Chen K., Pipe J.G., Maze S., Belden C., Maarouf C.L., Thiyyagura P., Mo H. (2012). Cerebral Blood Flow in Alzheimer’s Disease. Vasc. Health Risk Manag..

[37] Goldsmith H.S. (2022). Alzheimer’s Disease: A Decreased Cerebral Blood Flow to Critical Intraneuronal Elements Is the Cause. J. Alzheimers Dis..

[38] Fu T., Liu L., Huang X., Zhang D., Gao Y., Yin X., Lin H., Dai Y., Wu X. (2022). Cerebral Blood Flow Alterations in Migraine Patients with and without Aura: An Arterial Spin Labeling Study. J. Headache Pain..

[39] Honda M., Ichibayashi R., Yokomuro H., Yoshihara K., Masuda H., Haga D., Seiki Y., Kudoh C., Kishi T. (2016). Early Cerebral Circulation Disturbance in Patients Suffering from Severe Traumatic Brain Injury (TBI): A Xenon CT and Perfusion CT Study. Neurol. Med. Chir..

[40] Liu M., He E., Fu X., Gong S., Han Y., Deng F. (2022). Cerebral Blood Flow Self-Regulation in Depression. J. Affect. Disord..

[41] Mason B.N., Russo A.F. (2018). Vascular Contributions to Migraine: Time to Revisit?. Front. Cell. Neurosci..

[42] Anwar H.A., Ramya Krishna M., Sadiq S., Ramesh Kumar R., Venkatarathnam V., Saikiran G. (2022). A Study to Evaluate Neurovascular Conflict of Trigeminal Nerve in Trigeminal Neuralgia Patients with the Help of 1.5 T MR Imaging. Egypt. J. Radiol. Nucl. Med..

[43] Hannan C., Shoakazemi A., Quigley G. (2018). Microvascular Decompression for Trigeminal Neuralgia: A Regional Unit’s Experience. Ulst. Med. J..

[44] de Liyis B.G., Arini A.A.I.K., Karuniamaya C.P., Pramana N.A.K., Tini K., Widyadharma I.P.E., Setyopranoto I. (2024). Risk of Intracranial Hemorrhage in Brain Arteriovenous Malformations: A Systematic Review and Meta-Analysis. J. Neurol..

[45] Morgan M.K., Davidson A.S., Assaad N.N.A., Stoodley M.A. (2017). Critical Review of Brain AVM Surgery, Surgical Results and Natural History in 2017. Acta Neurochir..

[46] Panigrahy D., Gilligan M.M., Serhan C.N., Kashfi K. (2021). Resolution of Inflammation: An Organizing Principle in Biology and Medicine. Pharmacol. Ther..

[47] Wang L., Cheng C.K., Yi M., Lui K.O., Huang Y. (2022). Targeting Endothelial Dysfunction and Inflammation. J. Mol. Cell. Cardiol..

[48] Halade G.V., Lee D.H. (2022). Inflammation and Resolution Signaling in Cardiac Repair and Heart Failure. EBioMedicine.

[49] Li M., Hou Q., Zhong L., Zhao Y., Fu X. (2021). Macrophage Related Chronic Inflammation in Non-Healing Wounds. Front. Immunol..

[50] Fahed G., Aoun L., Bou Zerdan M., Allam S., Bou Zerdan M., Bouferraa Y., Assi H.I. (2022). Metabolic Syndrome: Updates on Pathophysiology and Management in 2021. Int. J. Mol. Sci..

[51] Mohammed S., Thadathil N., Selvarani R., Nicklas E.H., Wang D., Miller B.F., Richardson A., Deepa S.S. (2021). Necroptosis Contributes to Chronic Inflammation and Fibrosis in Aging Liver. Aging Cell.

[52] Jin L., Yu B., Armando I., Han F. (2021). Mitochondrial DNA-Mediated Inflammation in Acute Kidney Injury and Chronic Kidney Disease. Oxid. Med. Cell Longev..

[53] DeMaio A., Mehrotra S., Sambamurti K., Husain S. (2022). The Role of the Adaptive Immune System and T Cell Dysfunction in Neurodegenerative Diseases. J. Neuroinflamm..

[54] Greten F.R., Grivennikov S.I. (2019). Inflammation and Cancer: Triggers, Mechanisms and Consequences. Immunity.

[55] Majno G., Joris I. (2004). Cells, Tissues, and Disease: Principles of General Pathology.

[56] Feehan K.T., Gilroy D.W. (2019). Is Resolution the End of Inflammation?. Trends Mol. Med..

[57] Furman D., Campisi J., Verdin E., Carrera-Bastos P., Targ S., Franceschi C., Ferrucci L., Gilroy D.W., Fasano A., Miller G.W. (2019). Chronic Inflammation in the Etiology of Disease across the Life Span. Nat. Med..

[58] Kotas M.E., Medzhitov R. (2015). Homeostasis, Inflammation, and Disease Susceptibility. Cell.

[59] García-Escobar A., Vera-Vera S., Tébar-Márquez D., Rivero-Santana B., Jurado-Román A., Jiménez-Valero S., Galeote G., Cabrera J.-Á., Moreno R. (2023). Neutrophil-to-Lymphocyte Ratio an Inflammatory Biomarker, and Prognostic Marker in Heart Failure, Cardiovascular Disease and Chronic Inflammatory Diseases: New Insights for a Potential Predictor of Anti-Cytokine Therapy Responsiveness. Microvasc. Res..

[60] Zhang H., Dhalla N.S. (2024). The Role of Pro-Inflammatory Cytokines in the Pathogenesis of Cardiovascular Disease. Int. J. Mol. Sci..

[61] Kirabo A., Fontana V., de Faria A.P., Loperena R., Galindo C.L., Wu J., Bikineyeva A.T., Dikalov S., Xiao L., Chen W. (2014). DC Isoketal-Modified Proteins Activate T Cells and Promote Hypertension. J. Clin. Investig..

[62] Colonna M., Butovsky O. (2017). Microglia Function in the Central Nervous System During Health and Neurodegeneration. Annu. Rev. Immunol..

[63] Thakur S., Dhapola R., Sarma P., Medhi B., Reddy D.H. (2023). Neuroinflammation in Alzheimer’s Disease: Current Progress in Molecular Signaling and Therapeutics. Inflammation.

[64] Wolf D., Ley K. (2019). Immunity and Inflammation in Atherosclerosis. Circ. Res..

[65] The Emerging Risk Factors Collaboration (2010). C-Reactive Protein Concentration and Risk of Coronary Heart Disease, Stroke, and Mortality: An Individual Participant Meta-Analysis. Lancet.

[66] Thackeray J.T., Hupe H.C., Wang Y., Bankstahl J.P., Berding G., Ross T.L., Bauersachs J., Wollert K.C., Bengel F.M. (2018). Myocardial Inflammation Predicts Remodeling and Neuroinflammation After Myocardial Infarction. J. Am. Coll. Cardiol..

[67] Akiyama H., Barger S., Barnum S., Bradt B., Bauer J., Cole G.M., Cooper N.R., Eikelenboom P., Emmerling M., Fiebich B.L. (2000). Inflammation and Alzheimer’s Disease. Neurobiol. Aging.

[68] Roe K. (2021). An Inflammation Classification System Using Cytokine Parameters. Scand. J. Immunol..

[69] Scheitz J.F., Sposato L.A., Schulz-Menger J., Nolte C.H., Backs J., Endres M. (2022). Stroke–Heart Syndrome: Recent Advances and Challenges. J. Am. Heart Assoc..

[70] Hurskainen M., Tynkkynen J., Eskola M., Hernesniemi J. (2022). Incidence of Stroke and Mortality Due to Stroke after Acute Coronary Syndrome. J. Stroke Cerebrovasc. Dis..

[71] George M.S., Aston-Jones G. (2010). Noninvasive Techniques for Probing Neurocircuitry and Treating Illness: Vagus Nerve Stimulation (VNS), Transcranial Magnetic Stimulation (TMS) and Transcranial Direct Current Stimulation (tDCS). Neuropsychopharmacology.

[72] Johnson R.L., Wilson C.G. (2018). A Review of Vagus Nerve Stimulation as a Therapeutic Intervention. J. Inflamm. Res..

[73] Zhang Y., Popović Z.B., Bibevski S., Fakhry I., Sica D.A., Van Wagoner D.R., Mazgalev T.N. (2009). Chronic Vagus Nerve Stimulation Improves Autonomic Control and Attenuates Systemic Inflammation and Heart Failure Progression in a Canine High-Rate Pacing Model. Circ. Heart Fail..

[74] Borovikova L.V., Ivanova S., Zhang M., Yang H., Botchkina G.I., Watkins L.R., Wang H., Abumrad N., Eaton J.W., Tracey K.J. (2000). Vagus Nerve Stimulation Attenuates the Systemic Inflammatory Response to Endotoxin. Nature.

[75] Matteoli G., Gomez-Pinilla P.J., Nemethova A., Giovangiulio M.D., Cailotto C., van Bree S.H., Michel K., Tracey K.J., Schemann M., Boesmans W. (2014). A Distinct Vagal Anti-Inflammatory Pathway Modulates Intestinal Muscularis Resident Macrophages Independent of the Spleen. Gut.

[76] Diedrich A., Urechie V., Shiffer D., Rigo S., Minonzio M., Cairo B., Smith E.C., Okamoto L.E., Barbic F., Bisoglio A. (2021). Transdermal Auricular Vagus Stimulation for the Treatment of Postural Tachycardia Syndrome. Auton. Neurosci..

[77] Chapleau M.W., Rotella D.L., Reho J.J., Rahmouni K., Stauss H.M. (2016). Chronic Vagal Nerve Stimulation Prevents High-Salt Diet-Induced Endothelial Dysfunction and Aortic Stiffening in Stroke-Prone Spontaneously Hypertensive Rats. Am. J. Physiol.-Heart Circ. Physiol..

[78] Stavrakis S., Humphrey M.B., Scherlag B.J., Hu Y., Jackman W.M., Nakagawa H., Lockwood D., Lazzara R., Po S.S. (2015). Low-Level Transcutaneous Electrical Vagus Nerve Stimulation Suppresses Atrial Fibrillation. J. Am. Coll. Cardiol..

[79] Stavrakis S., Humphrey M.B., Scherlag B., Iftikhar O., Parwani P., Abbas M., Filiberti A., Fleming C., Hu Y., Garabelli P. (2017). Low-Level Vagus Nerve Stimulation Suppresses Post-Operative Atrial Fibrillation and Inflammation. JACC Clin. Electrophysiol..

[80] Yap J.Y.Y., Keatch C., Lambert E., Woods W., Stoddart P.R., Kameneva T. (2020). Critical Review of Transcutaneous Vagus Nerve Stimulation: Challenges for Translation to Clinical Practice. Front. Neurosci..

[81] Chinda K., Tsai W.-C., Chan Y.-H., Lin A.Y.-T., Patel J., Zhao Y., Tan A.Y., Shen M.J., Lin H., Shen C. (2016). Intermittent Left Cervical Vagal Nerve Stimulation Damages the Stellate Ganglia and Reduces the Ventricular Rate during Sustained Atrial Fibrillation in Ambulatory Dogs. Heart Rhythm..

[82] Guo B., Zhang M., Hao W., Wang Y., Zhang T., Liu C. (2023). Neuroinflammation Mechanisms of Neuromodulation Therapies for Anxiety and Depression. Transl. Psychiatry.

[83] Blake M.R., Gardner R.T., Jin H., Staffenson M.A., Rueb N.J., Barrios A.M., Dudley G.B., Cohen M.S., Habecker B.A. (2022). Small Molecules Targeting PTPσ-Trk Interactions Promote Sympathetic Nerve Regeneration. ACS Chem. Neurosci..

[84] Sepe J.J., Gardner R.T., Blake M.R., Brooks D.M., Staffenson M.A., Betts C.B., Sivagnanam S., Larson W., Kumar S., Bayles R.G. (2022). Therapeutics That Promote Sympathetic Reinnervation Modulate the Inflammatory Response After Myocardial Infarction. JACC. Basic. Transl. Sci..

[85] Qian K., Song L., Guo J.-M., Fu D., Shi J., Ma Y., Ge Z.-J., Li L., Zhang S.-Q. (2024). Baicalin Improves Isoproterenol-Induced Cardiac Remodeling by Regulating the Nrf2-Dependent Signaling Pathway. BMC Cardiovasc. Disord..

[86] Hanna A., Frangogiannis N.G. (2020). Inflammatory Cytokines and Chemokines as Therapeutic Targets in Heart Failure. Cardiovasc. Drugs Ther..

[87] Coveney S., McCabe J.J., Murphy S., O’Donnell M., Kelly P.J. (2020). Anti-inflammatory Therapy for Preventing Stroke and Other Vascular Events after Ischaemic Stroke or Transient Ischaemic Attack. Cochrane Database Syst. Rev..

[88] Lui A., Alzayat O., Do T., Perekopskiy D., Gann M., Elgokhy T.S., Gao J., Liu D. (2022). Multi-Targeted Anti-Inflammatory Drugs for the Treatment of Neurological Disorders. Neural Regen. Res..

[89] Ozben T., Ozben S. (2019). Neuro-Inflammation and Anti-Inflammatory Treatment Options for Alzheimer’s Disease. Clin. Biochem. Alzheimer’s Dis. Mak. Point.

[90] Su W.-J., Hu T., Jiang C.-L. (2024). Cool the Inflamed Brain: A Novel Anti-Inflammatory Strategy for the Treatment of Major Depressive Disorder. Curr. Neuropharmacol..

[91] Zhang W., Zhang S., Deng Y., Wu S., Ren J., Sun G., Yang J., Jiang Y., Xu X., Wang T.-D. (2021). Trial of Intensive Blood-Pressure Control in Older Patients with Hypertension. N. Engl. J. Med..

[92] Carcel C., Haghdoost F., Shen J., Nanda P., Bai Y., Atkins E., Torii-Yoshimura T., Clough A.J., Davies L., Cordato D. (2023). The Effect of Blood Pressure Lowering Medications on the Prevention of Episodic Migraine: A Systematic Review and Meta-Analysis. Cephalalgia Int. J. Headache.

[93] Jadhav A.P., Desai S.M., Jovin T.G. (2021). Indications for Mechanical Thrombectomy for Acute Ischemic Stroke: Current Guidelines and Beyond. Neurology.

[94] Yang L., Cheng H. (2022). Surgical Technique Management of Microvascular Decompression for Trigeminal Neuralgia. Ideggyogy. Szle..

[95] Cao Y., Redd M.A., Fang C., Mizikovsky D., Li X., Macdonald P.S., King G.F., Palpant N.J. (2023). New Drug Targets and Preclinical Modelling Recommendations for Treating Acute Myocardial Infarction. Heart Lung Circ..

